# Supplementary material for: Identification and functional analysis of non-coding regulatory small RNA FenSr3 in Bacillus amyloliquefaciens LPB-18
Source: PeerJ. 2023 May 15;11:e15236. doi: 10.7717/peerj.15236 (PMC10194069; doi:10.7717/peerj.15236)
Supplement: Supplemental Information 4 [file peerj-11-15236-s004.zip › KO/CK-vs-T1_map/map04710.html]

KEGG PATHWAY: Circadian rhythm - Reference pathway


|  |  |
| --- | --- |
| **Circadian rhythm - Reference pathway** |  |

[
Pathway menu
| Organism menu
| Pathway entry
| Show description
| User data mapping
]

Circadian rhythm is an internal biological clock, which enables to sustain an approximately 24-hour rhythm in the absence of environmental cues. In mammals, the circadian clock mechanism consists of cell-autonomous transcription-translation feedback loops that drive rhythmic, 24-hour expression patterns of core clock components. The first negative feedback loop is a rhythmic transcription of period genes (PER1, PER2, and PER3) and chryptochrome genes (CRY1 and CRY2). PER and CRY proteins form a heterodimer, which acts on the CLOCK/BMAL1 heterodimer to repress its own transcription. PER and CRY proteins are phosphorylated by casein kinase epsilon (CKIepsilon), which leads to degradation and restarting of the cycle. The second loop is a positive feedback loop driven by the CLOCK/BMAL1 heterodimer, which initiates transcription of target genes containing E-box cis-regulatory enhancer sequences.

|  |  |  |
| --- | --- | --- |
| Reference pathway | 184% 150% 122% 100% 82% 67% 55% | 图片下载 |
